# Supportive care of female hormones in brain health: what and how?

**DOI:** 10.3389/fphar.2024.1403969

**Published:** 2024-07-24

**Authors:** Afang Zhu, Shujia Song, Lijian Pei, Yuguang Huang

**Affiliations:** Department of Anesthesiology, Peking Union Medical College Hospital, Chinese Academy of Medical Sciences, Beijing, China

**Keywords:** estrogen, progesterone, depression, pain, cachexia, social interaction

## Abstract

Female hormones, functioning as neuroactive steroids, are utilized beyond menopausal hormone therapy. The rapid onset of allopregnanolone analogs, such as brexanolone and zuranolone, in treating depression, and the effectiveness of megestrol acetate in addressing appetite and weight gain, prompted the Food and Drug Administration to authorize the use of progesterone for treating postpartum depression and cancer-related cachexia. Progesterone has also been found to alleviate neuropathic pain in animal studies. These off-label applications offer a promising option for patients with advanced cancer who often experience various mood disorders such as depression, persistent pain, social isolation, and physical complications like cachexia. These patients have shown low tolerance to opioids and mood-regulating medications. However, the potential risks and uncertainties associated with hormone therapy treatment modalities can be daunting for both patients and medical professionals. This review aims to offer a comprehensive understanding of the non-reproductive functions and mechanisms of female hormones in brain health.

## Introduction

Patients with cancer often experience refractory pain. It is projected that there will be over 28.4 million new cases of cancer globally in 2040 (Sung et al., 2021). 39.3% of cancer survivors experience pain, and this percentage increases to 66.4% among individuals with advanced cancer (Van Den Beuken-Van Everdingen et al., 2016). Nevertheless, the effectiveness and acceptance of opioids for these patients are unsatisfactory, not to mention their associated side effects with long-term use. Additionally, the coexistence of pain and depression is a prevalent phenomenon among this population (Adam et al., 2021), often accompanied by a tendency to passively seek medical attention and exhibit suboptimal nutritional status (Adams et al., 2018; Law, 2022). Hence, our objective is to find a supplementary medication that can effectively reduce the dependence on opioids while also improving the emotional wellbeing and physical status of these patients. Benefiting from a long-term focus on women’s health, our attention is directed towards neuroactive steroids, especially female hormones, and their potential non-reproductive functions in the aforementioned patients. Neuroactive steroids refer to steroids that target neurons and glial cells. They include hormonal steroids produced by the peripheral glands, neurosteroids synthesized by neurons and glial cells, and synthetic steroids that alter the functioning of the nervous system.

The brain and the ovary are integral components of the neuroendocrine system and are intricately interconnected (Hogervorst et al., 2022). Hormones produced by the ovaries have the capacity to impact brain function. For instance, a reduction in estrogen levels results in a slowing down of brain function and a hastening of the aging process. This process may also contribute to the formation of amyloid plaques, which are linked to the pathogenesis of Alzheimer’s disease (Oveisgharan et al., 2023). Women are at an increased risk of developing dementia if they undergo oophorectomy before reaching menopause (Uldbjerg et al., 2022). In addition to affecting cognitive function, the hormones produced by the ovaries also play a role in mental health, including depression and social interaction. For instance, allopregnanolone (AP) analogs have been approved by the Food and Drug Administration (FDA) for the rapid management of postpartum depression (PPD) (Scott, 2019), and the significance of oxytocin in social dysfunction is widely acknowledged (Jones et al., 2017). However, hormonal therapy is still predominantly utilized for the relief of symptoms such as menopausal hot flashes up to the present time.

Females are disproportionately affected by numerous illnesses affecting the brain, and emerging evidence suggests a high incidence of depression in endocrine transition phases like perimenopause and postpartum (Mauvais-Jarvis et al., 2020). Reduction and fluctuation of female hormones can, in part, explain the etiology of depression, pain, and social interaction, but the effectiveness of supplementation in prevention or treatment remains incompletely understood. Furthermore, there are numerous concerns regarding the potential of hormone replacement therapy (HRT) to trigger tumor recurrence or metastasis. Thus, we review the non-reproductive effects of female hormones on pain, depression, social interaction, cachexia, and their underlying mechanisms and treatment implications. It is expected that medical professionals will be able to provide evidence-based and alternative approaches for managing patients with relevant medical conditions.

### Female hormones and depression

More than 350 million people suffer from depression, and women exhibit a higher prevalence of major depression compared to men, with a global annual prevalence rate of 5.5% among women and 3.2% among men (Whiteford et al., 2013). Furthermore, over 70% of depression in women occurs during the menopausal period, and one out of every three women will experience notable psychological changes during the menopausal transition (MT) (Maki et al., 2019). Studies have shown that the incidence of depression is more than fourfold higher during the MT compared to the pre-menopausal period. For women who have a history of depression, the risk even increases to 60% (Freeman et al., 2006). In addition, women are not only at an increased risk of depression and anxiety during perimenopause and MT, but also experience depressive symptoms of higher severity compared to pre- and postmenopause.

Estrogen fluctuations and loss have been shown to be closely related to depression in perimenopausal women. Women who undergo bilateral oophorectomy without receiving estrogen replacement therapy (ERT) are more likely to experience major depression (Stuursma et al., 2022). Therefore, the estrogen withdrawal theory is proposed. The theory suggests that a rapid decrease in estradiol (E2) secretion, the most biologically active estrogen, is associated with an increase in mood disorders during this period, resulting in new or recurrent depressive symptoms. However, the antidepressant effect of estrogen therapy depends on various factors, including the type of estrogen, duration of treatment, dosage, time elapsed after ovariectomy, and age. For instance, estrogen has maximal protective benefits on cognition in women when it is initiated closely in time to natural or surgical menopause (Maclennan et al., 2006). However, treatment begun decades after menopause does not have the same effect. This means that perimenopausal women are more responsive to ERT than postmenopausal women. Similarly, in young rats, 3 months after ovariectomy, the acute injection of E2 failed to produce an antidepressant-like action. However, if E2 was administered 1 or 3 weeks after ovariectomy, it produced clear antidepressant-like effects (Estrada-Camarena et al., 2011). Interestingly, another estrogen, EE2, was able to induce antidepressant-like actions in young rats even 4 months after ovariectomy (Estrada-Camarena et al., 2011). This suggests that each estrogen has a specific time frame in which it is effective.

Though there is some evidence that estrogen has antidepressant effects similar in magnitude to those observed with classic antidepressant agents when administered to depressed perimenopausal women, estrogen is still not FDA-approved to treat mood disturbances. Hormone therapy is FDA-approved for the treatment of hot flashes and vaginal dryness, but it is not approved for the treatment or prevention of mood disturbances. However, the use of time-limited ERT or HT is reasonable in perimenopausal women experiencing physical symptoms of menopause such as vasomotor symptoms and vaginal dryness, in addition to depression.

Besides menopausal depression, women also suffer from postpartum depression (PPD), which typically occurs 2 weeks after delivery and can last for 3–6 months or even longer. It is estimated that 7% of women experience an episode of major depression in the first 3 months following delivery, and the prevalence increases to 20% when episodes of minor depression are also taken into account (Gavin et al., 2005). The average prevalence of PPD worldwide is 17.22%, while in China it ranges from 7.3% to 37.14% (Wang et al., 2021). PPD not only causes damage to the mother but also affects the growth and development, emotions, behavior, and intelligence quotient of the infant. It increases the risk of depressive disorder, attention deficit hyperactivity disorder, and autism spectrum disorder in children (Becker et al., 2016).

During late pregnancy, estrogen levels are 49 times higher than the peak of the menstrual cycle, and progesterone levels are 9–10 times higher than the peak of the menstrual cycle. Both hormones return to pre-pregnancy levels shortly after the delivery of the fetus (Bloch et al., 2003; Mccoy et al., 2003; Wable et al., 2015). But the results regarding whether hormones contribute to PPD have been mixed. Absolute levels of estrogen and progesterone may not differ in women with maternal depression, but women who develop PPD may be more sensitive to fluctuations in these hormones. Estrogen and progesterone withdrawal can lead to increased depression in certain women, especially those who have previously experienced PPD (Bloch et al., 2000). However, there is no convincing evidence that women who develop PPD experience more rapid postpartum hormone withdrawal, have lower concentrations of reproductive hormones during the postpartum period, or experience greater reductions in hormone levels from pregnancy to the postpartum period compared to women without PPD. A double-blind, placebo-controlled study of 61 women with PPD that started within 3 months after delivery revealed that women who were treated with transdermal estradiol (E2) over placebo showed significant improvement. While another pilot RCT involving 30 women with PPD treated with sublingual E2 did not show significant efficacy compared to the placebo (Kettunen et al., 2022). It is worth noting that almost half of the women in both groups were also taking antidepressant medication. In addition, the adverse effects of estrogen supplementation are related to different absorption pathways. Oral estrogen collects in the hepatic portal vein and is inactivated in the liver, greatly reducing the efficiency of estrogen. Estrogen metabolism in the liver may impact the balance of coagulation and anticoagulation systems, leading to an increase in the concentration of prothrombin fragments in patients and a decrease in the concentration of antithrombin. This can result in resistance to activated protein C and elevate the risk of thrombosis. While transdermal absorption can bypass the liver’s first-pass effect of oral estrogen, and the dosage is typically lower than the oral dose. This results in a stable concentration of estrogen, making it more suitable for long-term use. It should be noted that, estrogen therapy can hinder lactation, which can impact the choice of postpartum breastfeeding for patients with depression.

Alterations in progesterone metabolites, including allopregnanolone (AP), have also been suggested to play a role in PPD, though conflicting results have been reported. For example, Osborne et al. found that low levels of AP during pregnancy predicted subsequent PPD (Osborne et al., 2017), while Deligiannidis et al. showed higher concentrations of AP in the peripartum plasma of women who developed PPD (Deligiannidis et al., 2019). Clinical trials have demonstrated efficacy of AP supplement in treating PPD.

For the poor bioavailability of exogenously administered AP, trials have relied on small molecules similar to AP. Among these, γ-aminobutyric acid (GABA)-A receptor selective positive allosteric modulators such as brexanolone (SAGE-547), zuranolone (SAGE-217), and ganaxolone are commonly studied and have shown great potential in treating PPD (Table 1). A double-blind, placebo-controlled RCT with 10–11 females with severe PPD per group, followed by two larger multi-center RCTs involving a total of 246 patients, demonstrated that a 60-hour infusion of brexanolone (SAGE-547, a synthetic formulation of AP) led to significant reductions in depressive symptoms among women with severe PPD (Meltzer-Brody et al., 2018). On 19 March 2019, brexanolone was approved by the FDA for the treatment of PPD (Wisner et al., 2019), making it the first specific treatment for PPD. Despite the bright future of brexanolone due to its rapid onset, the need for continuous infusion under medical supervision and its high cost have prompted researchers to develop other drugs. It is worth noting that as the first-line treatments for PPD, SSRIs take several weeks to show effectiveness, while brexanolone has rapid antidepressant effects, making it potentially suitable for the critical early stages of PPD (Meltzer-Brody et al., 2018).

**TABLE 1:** Comparisons of various AP analogs in treating depression.

Compound	Dosage form	Indication	Advantage	Mechanism	Attention	Dosage used	Others
Brexanolone, SAGE-547	Intravenous	Moderate and severe PPD	Rapid onset (60 h) of action	Positive allosteric modulators of GABA-AR.	Need to be under thorough medical supervision because of the risk of severe sedation, hypnosis, loss of consciousness and profound respiratory arrest. Long hours of administration time, and the high cost of $34,000. Commons side effects included flushing or hot flashes, dry mouth, dizziness and somnolence	Continous infusion at 60 or 90 mcg/kg/h for 60 h	
Zuranolone, SAGE-217	Oral	Major depressive disorder and severe PPD	Rapid onset (day 3) and persist up to 45 days in PPD. Symptom reduction by day 15 with 50 mg daily in major depressive disorder	Enhance inhibitory GABAergic signaling by increasing synaptic and extrasynaptic GABA-A activity and regulation of GABA-AR expression	Common side effects reported were somnolence, dizziness, balance disorder, diplopia, dysarthria and gait impairments	30 mg once-daily, for 14 days; 50 mg for severe PPD; the maximum tolerated dose was 55 mg. Not suitable for long-term use	Approved by FDA for PPD. In Phase III clinical trials for MDD, and insomnia. In Phase II clinical studies for bipolar depression, essential tremor, and Parkinson’s disease. In the preclinical trial stage for dyskinesias
Ganaxolone	Both intravenous and oral	Phase II and Phase III studies are ongoing and planned where ganaxolone is being developed for PPD (NCT03460756), pharmacoresistant status epilepticus, and several rare, treatment-resistant genetic epilepsies (e.g., CDLK5 deficiency disorder, NCT03572933, NCT05249556)	Great therapeutic potential in treating epilepsy	An extrasynaptic and synaptic GABA-AR positive allosteric modulator	Mild adverse events including somnolence, fatigue, dizziness, and headache	1,500 mg/day for 6 weeks	Preclinical efficacy in ameliorating social isolation
NORA520	Oral	Phase II study are ongoing in female adults with severe PPD (NCT06285916)	Active moiety, rapid onset of action, enhancing oral absorption and proloing half-life	Structurally identical to allopregnanolone, binds to extrasynaptic GABAA receptor and increases tonic GABAergic current			
LYT-300	Oral	Phase IIa clinical trial are ongoing. Targeted for treating anxiety, mood disorders and Fragile X-associated Tremor/Ataxia Syndrome (NCT05129865)	High bioavailability, approximately nine-fold greater than that of orally administered AP.	Positive allosteric modulators of GABA-AR.			
SGE-516	Both intravenous and oral	Preclinical: Detect compounds with anticonvulsant and mood-enhancing efficacy	Acute treatment exhibits robust therapeutic effects in preclinical PPD models	Neuroactive steroid; increase allopregnanolone levels in the basolateral amygdala			
SAGE-324	Oral	A phase IIb KINETIC 2 trial. Efficacious in reducing essential tremor (NCT04305275, NCT05173012, NCT05366751)		A neuroactive steroid GABAkine and a delta-preferring GABAkine	Common side effects reported were somnolence, dizziness, balance disorder, diplopia, dysarthria and gait impairments	Uptitration from 15 mg to 60 mg, daily at night over 28 days	

PPD: postpartum depression; GABA-AR: γ-aminobutyric acid type A receptor; MDD: major depression disorder.

SAGE-217 was orally bioavailable, and the maximum tolerated dose of the oral solution formulation was established as a single dose of 55 mg and multiple doses of 30 mg (Hoffmann et al., 2020). There have been two phase III trials of zuranolone for PPD and both demonstrated efficacy by day 15 of treatment. In both studies, the drug was well tolerated, with a dosage of 30 mg/day in the first phase III PPD study (Deligiannidis et al., 2021) and 50 mg/day in the second phase III PPD study (Deligiannidis et al., 2023). Reductions in depressive symptoms were observed by day 3 and sustained at all measured time points through day 45 in both studies. The FDA approved the drug for PPD but did not approve zuranolone for major depressive disorder at the time. A double-blind, placebo-controlled RCT recruited patients with major depressive disorder. They received zuranolone at doses of 20 mg, 30 mg, or a placebo for 14 days. No significant improvement was observed at any measured time point between zuranolone 20 mg and placebo. Improvement *versus* placebo was significant on days 3, 8, and 12 for the zuranolone 30 mg group, while it was similar between day 15 and day 182 (Clayton et al., 2023).

Ganaxolone, being developed for treating PPD, is a 3β-methylated synthetic analog of AP with effects on extrasynaptic and synaptic GABA-A receptors. Clinical trials investigating the treatment of PPD (NCT03460756; NCT03228394) are currently in progress. In addition, there is an open-label, uncontrolled pilot study indicating that adjunctive ganaxolone seems to have antidepressant effects but causes sedation. In this study, 10 post-menopausal women with persistent depression received open-label ganaxolone (225 mg bid, increased to 450 mg bid if tolerated) for 8 weeks, followed by a 2-week taper (Dichtel et al., 2020).

There has long been evidence that oral contraceptives (OCPs) have the ability to affect mood. According to two large prospective studies that looked at the medical records of over a million Danish women over a 10-year period, OCPs use is associated with increased risk for depression diagnosis, antidepressant treatment, and suicidal acts (Skovlund et al., 2016; Skovlund et al., 2018). In contrast, a smaller cross-sectional study describes an increased risk of mood disorders with progestin-only OCPs but decreased risk of mood disorders when combined estrogen-progestin OCPs (Svendal et al., 2012). However, a comprehensive analysis of 26 trials involving progestin-only OCPs found no consistent evidence of relationship with depressed symptoms; the majority of these studies were classified as low-quality or significantly biased (Worly et al., 2018). An even more complex picture is presented in an RCT with 178 women from a population sample, where OCPs are linked to an improvement in mood during the premenstrual phase but a worsening of mood symptoms in the intermenstrual period (Lundin et al., 2017). For example, women with polycystic ovarian syndrome have higher rates of depression and anxiety (Cooney et al., 2017). OCPs are associated with an improvement in depressive symptoms and health-related QoL (Dokras et al., 2016). Taken together, these complex data suggest that ovarian hormones can have significant effects on mood. However, they also indicate a complex underlying biology that is influenced by factors such as age, timing within the menstrual cycle, and the administration of hormones.

Emerging evidence suggests that sex steroid exposure and exogenous sex steroid use shape the female brain, and might contribute to the risk for Alzheimer’s disease later in life. Depression is also a known risk factor for Alzheimer’s disease (Sáiz-Vázquez et al., 2021; Jett et al., 2022). However, the influence of depression emerging with hormonal changes of pregnancy and menopause, and HT on Alzheimer’s disease risk is rarely studied and remains controversial. Compared with oral E2-based types, which present with an increased risk of Alzheimer’s disease, vaginal and transdermal E2-based HT are effective and safer in reducing the risk of Alzheimer’s disease (Savolainen-Peltonen et al., 2019; Kim et al., 2021). More research is needed to establish the link among subtypes of depression, E2, and Alzheimer’s disease.

### Mechanisms

Estrogen can affect depressive disorders by regulating a variety of neurotransmitters, including 5-hydroxytryptamine (5-HT), dopamine (DA), glutamate, and GABA. It increases the synthesis and availability of 5-HT in the dorsal raphe nucleus, which plays a role in emotional regulation through the activation of 5-HT receptors, particularly the 5-HT1A receptor (Gundlah et al., 2002; Hiroi et al., 2006). Activation of the 5-HT1A receptor has been found to have antidepressant and anti-anxiety effects. Estrogen also selectively increases 5-HTA density in brain regions that contain ER, such as the thalamus, preoptic area, and amygdala (Moses-Kolko et al., 2008). Estrogen can also regulate the serotonin transporter, which reuptakes 5-HT from the synaptic cleft back into the presynaptic neuron. The reactivity of 5-HT decreases during premenstrual, perimenopausal, and postmenopausal periods, making women more susceptible to depression. However, it can be restored through E2 treatment. Estrogen can also inhibit the degradation of monoamine oxidase and promote an increase in tryptophan hydroxylase levels, thereby increasing the concentration of 5-HT in the blood plasma.

Estrogen can also exert antidepressant effects by affecting DA metabolism through promoting the synthesis and release of DA. In a model of perimenopausal depression, female rats were subjected to ovariectomy. The study found that the levels of DA in the striatum of ovariectomized rats were significantly lower compared to non-ovariectomized rats. This suggests that estrogen can increase the levels of DA in the striatum and even enhance its release in the hypothalamus and anterior pituitary, thereby alleviating depressive symptoms. In addition, estrogen can upregulate the expression and function of DA receptors. Chronic E2 treatment increases DA receptor density in the striatum and nucleus accumbens (Landry et al., 2002). The levels of 5-HT and DA in the serum of patients with PPD were also found to be decreased compared to patients without PPD. These levels were negatively correlated with the Edinburgh Postnatal Depression Scale score. In addition, estrogen can have a positive regulatory effect on NMDA receptors in postsynaptic membranes, thereby enhancing the release of glutamate at synapses. It can also facilitate the transmission of glutamatergic and DAergic neurons by promoting the influx of Ca^2+^ (D’anglemont De Tassigny et al., 2009).

In addition, GABAergic neurons are also involved in PPD development. Enhancing the activity of neurons in the medial preoptic area ameliorates depressive-like behaviors in mice, while reducing their activity leads to the expression of these behaviors. A neuronal ensemble projecting to the ventral tegmental area mediates anhedonia, while another projecting to the periaqueductal gray mediates immobility. The two projections increase the activity of dopaminergic neurons in the ventral tegmental area and serotonergic neurons in the dorsal raphe, leading to higher dopamine and serotonin release. Thus, GABAergic neurons in the medial preoptic area mediate PPD (Tao et al., 2023).

There is mounting evidence that inflammation is closely linked to depression. Overexpression of proinflammatory cytokines in the brain can contribute to the development of anxiety and depression-like behaviors. Estrogen is a sex steroid hormone with anti-inflammatory effects and plays an important role in suppressing the inflammatory response in depression. Estrogen can reduce the inflammatory response activity of astrocytes and microglia, as well as decrease the release of inflammatory factors such as IL-6, TNF-α, and NO (Rachoń et al., 2002). In the mouse model of perimenopausal depression, which was constructed by performing ovariectomy, it was observed that the levels of inflammatory factors and nucleotide-binding and oligomerization domain-like receptor family 3 (NLRP3) inflammasome in the hippocampus of mice increased in the estrogen-deficient state. Additionally, the mice exhibited depression-like behavior (Wang et al., 2016). Estrogen and ERβ agonist supplementation can reduce the levels of inflammatory factors and the NLRP3 inflammasome in the hippocampus of mice, thereby improving the depression-like behavior of mice. Long-term use of HRT is effective in decreasing inflammation and increasing antioxidant contents in the serum of postmenopausal women (Jee et al., 2021).

Oxytocin (OT) has also been implicated in PPD for its functions in regulating emotion, stress, and maternal care. Low levels of oxytocin during pregnancy or postpartum have been suggested as a risk factor for PPD. However, supplementing OT in postpartum women may worsen mood (Mah, 2016). Interestingly, a recent study using a hormone-simulated pseudopregnancy model shows that estrogen withdrawal increases postpartum anxiety through OT plasticity, including changes in OT-immunoreactive cells, mRNA, and receptor density in the paraventricular hypothalamus and dorsal raphe nucleus (Hedges et al., 2021).

ER can be divided into nuclear receptors and membrane receptors. Nuclear receptors can be divided into estrogen receptor (ER) α and ERβ, and membrane receptors mainly include the G protein-coupled estrogen receptor (GPER). Studies have shown that ERβ plays an important role in regulating depressive-like behaviors. In the ERβ knockout mice, depression-like behavior was significantly increased, and estrogen had no significant effect. Studies in gonadectomized mice have shown that ERβ agonists can effectively reduce depression-like behaviors (Toufexis, 2007; Solomon et al., 2009). These results suggest that estrogen exerts an antidepressant effect through ERβ. Reduced levels of 5-HT and DA in the brain have been observed in ERβ knockout mice, indicating that ERβ plays a crucial role in the regulation of brain amines by estrogen. The rapid antidepressant effect of estrogen may be related to GPER, which is widely distributed in the central and peripheral nervous system, including the hippocampus, hypothalamus, midbrain, and spinal cord, in both female and male rats (Prossnitz et al., 2011; Lu et al., 2017). The selective GPER agonist G1 exerts an antidepressant effect similar to E2 in a mouse depression model, which can be inhibited by the selective GPER antagonist G15. This suggests that the activation of GPER may play a role in treating depression (Prossnitz et al., 2015).

Multiple antidepressant agents, antipsychotic drugs, and mood stabilizers have been shown to increase AP brain levels in animal studies (Jaworska-Feil et al., 2000; Marx et al., 2006). This suggests that enhancing AP could be a significant mechanism for the antidepressant effects of these drugs. Animal studies have found that selective serotonin reuptake inhibitors (SSRIs) increase the levels of AP in various areas of the rat brain. This has led to the hypothesis that depression may be associated with reduced AP levels in patients. It is further suggested that the administration of SSRIs could normalize AP levels in the brain of depressed patients, thereby improving depressive symptoms. This was verified by Uzunova et al., who measured the levels of AP in the cerebrospinal fluid (CSF) before and after 8–10 weeks of treatment with fluoxetine or fluvoxamine in patients with unipolar depression (Uzunova et al., 1998). Before initiating antidepressant pharmacotherapy, the concentrations of AP were found to be approximately 60% lower in depressed patients compared to age- and sex-matched non-psychiatric subjects. At the end of the treatment period, there was a normalization and re-increase of AP concentrations in the CSF. This increase was significantly correlated with the improvement of depressive symptoms, as measured by the Hamilton Rating Scale for Depression.

**FIGURE 1:**
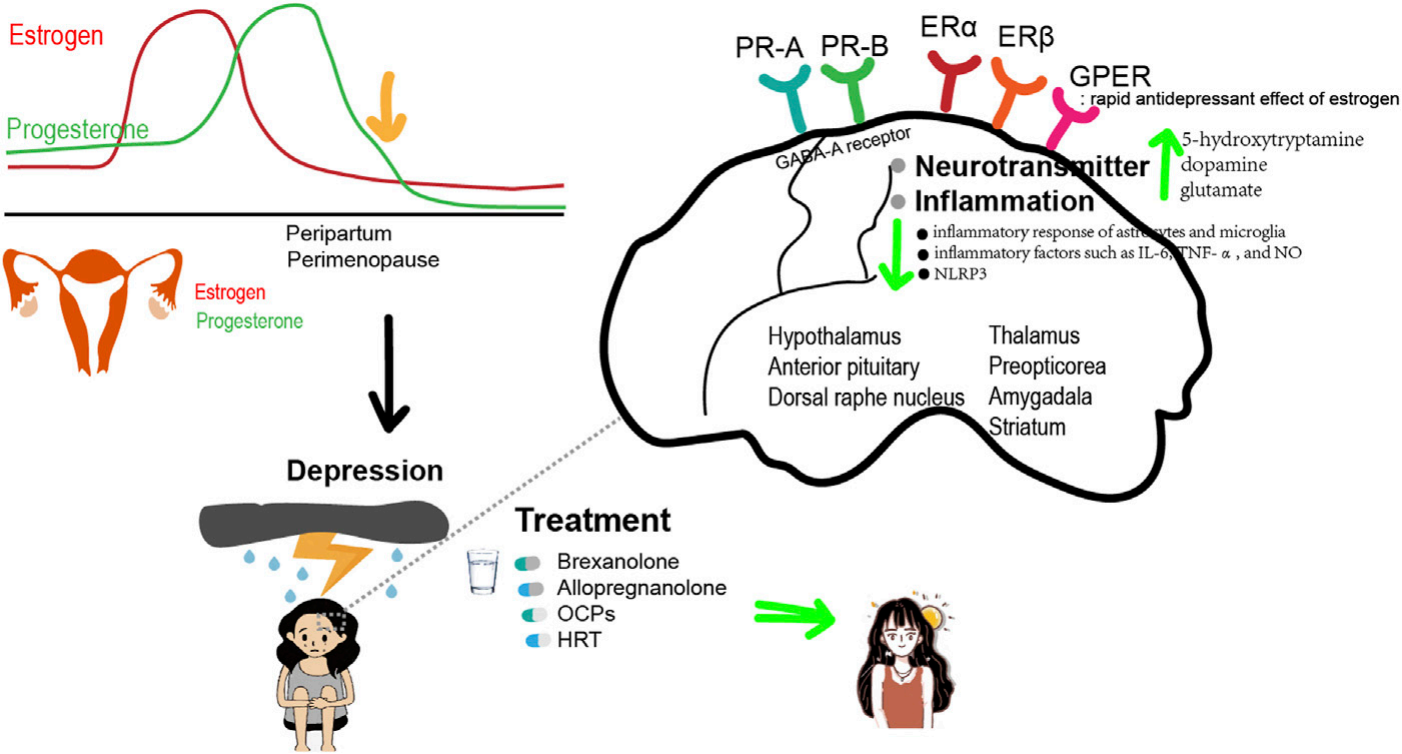
Estrogen and Progesterone relieve depression.

Progesterone is highly lipophilic and easily crosses the blood-brain barrier. As a result, concentrations of progesterone in certain regions of the brain can be higher than those measured in the serum. Similar to progesterone, AP and other progesterone metabolites also accumulate in the brain. In women, the highest concentrations of AP are found in the basal hypothalamus, followed by the substantia nigra and the amygdala. Functional brain imaging was introduced to further investigate the impact of AP on emotion regulation. A 3T functional magnetic resonance imaging (fMRI) was performed while participants engaged in the shifted-attention emotion appraisal task to investigate emotional regulation and processing. Compared to the placebo, administration of pregnenolone (400 mg) resulted in elevated AP levels (Sripada et al., 2013). These elevated levels were found to be associated with decreased activity in the amygdala and insula, but increased activity in the dorsal medial prefrontal cortex. Additionally, there was enhanced connectivity observed between the amygdala and the dorsal medial prefrontal cortex. Moreover, these effects on emotion-regulatory neurocircuits were associated with a reduction in self-reported anxiety in the 31 healthy male volunteers (Sripada et al., 2013). Notably, the amygdala and medial prefrontal cortex are rich in GABA-A receptors and endogenous AP, suggesting that AP could feasibly have a direct influence on the activity in these brain regions (Pirker et al., 2000). Others have speculated that PPD could be caused by a deficit in GABA-A receptor modulation rather than AP itself because AP is a strong allosteric modulator of the GABA-A subtype. This is corroborated by a study with knockout mice that showed anxiety- and depression-like behaviors after giving birth because they were unable to regulate the GABA-A receptor during the peripartum phase (Maguire et al., 2008). In female rats, AP injected intracerebroventricularly regulates GABA-A receptors to produce anxiolytic-like effects (Bitran et al., 1991). Distinct from other GABA-A positive allosteric modulators, such as benzodiazepines, AP in the basolateral amygdala enhances high-theta oscillations through delta-containing GABA-A receptors (Antonoudiou et al., 2022). Significantly, progesterone injection raises brain AP levels in both male and female rats, exhibiting anxiolytic-like effects that may be inhibited by GABA-A receptor inhibitors. According to animal studies, progesterone receptors (PR), specifically PR-A and PR-B, are strongly expressed in regions critical to cognitive function and emotional processing (Schumacher et al., 2014). These regions include the frontal cortex, thalamus, hippocampus, hypothalamus, and amygdala (Holder et al., 2015) (Figure 1).

### Progesterone and neuropathic pain

There have been some observational clinical studies indicating an association between neuroactive steroids and neuropathic pain in patients. Women with migraines presented significantly lower serum levels of pregnenolone and AP, which were negatively associated with the progression of migraines (Rustichelli et al., 2021a; Rustichelli et al., 2021b). Breast and ovarian cancer are hormone-dependent cancers, which means that they cannot grow in the absence of hormones. The two cancers that have high estrogen and low progesterone levels are prone to developing peripheral neuropathy and neuropathic pain (Mchann et al., 2021). In addition, there was a significant negative correlation between pain severity and daily changes in progesterone and testosterone (Schertzinger et al., 2018). Serum progesterone level showed a negative correlation with the pain score after cesarean section (Kashanian et al., 2019). Together, these observations led us to conclude that progesterone and AP may play a protective role in reducing pain severity. However, there are few clinical studies on exogenous or synthetic neuroprotective steroids as analgesics, which could be promising future treatment for neuropathic pain due to their high therapeutic potential.

### Mechanisms

AP was found to be increased in the central nervous system (CNS) in many pain models. In the sciatic nerve ligation rat model, the level of AP in the spinal cord of hyperalgesic rats was greater than that of the control animals (Kawano et al., 2011a). The analgesic effect of paroxetine is associated with increased levels of AP in the spine in the rat neuropathic pain model (Kawano et al., 2011b). Consistent with these results, several studies have confirmed that key enzymes important for progesterone and AP biosynthesis throughout the CNS, including cytochrome P450 side-chain-cleavage and 3α-hydroxysteroid oxido-reductase (3alpha-HSOR), are upregulated in the dorsal horn in chronic constriction injury (CCI) rats (Patte-Mensah et al., 2004; Meyer et al., 2008). Most importantly, the use of siRNA to knock down the expression of 3alpha-HSOR exacerbated thermal and mechanical pain perceptions (Patte-Mensah et al., 2010).

Several studies have suggested that progesterone is a promising agent for modulating mechanical and cold allodynia after spinal cord injury (Coronel et al., 2011b; Coronel et al., 2014; Coronel et al., 2016). Indeed, numerous studies in animal models of peripheral nerve injury have demonstrated the protective effect of progesterone and its metabolites. Exogenous progesterone and AP can alleviate allodynia in animal models of neuropathic pain, such as peripheral nerve injury, diabetic neuropathy, neuropathic pain caused by anticancer drugs, and spinal cord injury. AP treatment can effectively reduce the immunoreactivity and pro-inflammatory cytokine release of p-ERK and OX-42, and improve the mechanical hypersensitivity response in CCI rats (Huang et al., 2016). In a rat model of nerve crush injury, treatment with dihydroprogesterone (DHP) or progesterone was found to decrease the density of myelinated fibers, increase reelin mRNA levels, regulate biochemical parameters, and normalize thermal threshold (Roglio et al., 2008). In good agreement with these results, treatment with medroxyprogesterone acetate (MPA) led to a decrease in endogenous AP levels, rendering rats susceptible to neurologic changes caused by nerve injury (Huang et al., 2016). The use of AP in a diabetic neuropathy model could counteract the downregulation of the GABA-A receptor, which plays an inhibitory role in the neural circuits associated with neuropathic pain (Afrazi et al., 2014). Furthermore, AP significantly improved the hyperglycemia-induced hyperalgesia and motor dysfunction. Driving the synthesis of progesterone, AP and DHP in the sciatic nerve, the liver X receptor ligands could significantly increase the decrease in thermal threshold of diabetes-induced neuropathy. This reflects the critical protective effect of neuroactive steroids in diabetic neuropathic pain (Cermenati et al., 2010). In addition, AP could counteract the neurochemical, electrophysiological, and functional alterations in peripheral nerves and suppress anticancer drug-evoked painful neuropathy caused by oxaliplatin and vincristine (Meyer et al., 2010; Meyer et al., 2011; Taleb et al., 2017).

The mechanism of how progesterone and AP exert analgesic effects has not been fully clarified. The preclinical evidence has revealed several potential targets of progesterone and its metabolites for preventing neuropathic pain in various animal models. It is well known that alterations in the expression of NMDAR subunits in the spinal cord are significantly associated with abnormal pain processing. Remarkably, progesterone prevents upregulation of NMDAR subunits and PKCγ mRNAs after spinal cord injury (Coronel et al., 2011a; Coronel et al., 2011b). Additionally, progesterone may prevent allodynia by modulating neuroinflammatory responses. Experimental animals with neuropathic pain that received progesterone showed decreased mRNA levels of proinflammatory factors such as IκB-α, IL-1β, IL-6, and TNF-α (Coronel et al., 2014; Coronel et al., 2016). Additionally, there was a decrease in COX-2, iNOS, and OX-42 positive cells compared to the control group (Coronel et al., 2014). Furthermore, transient receptor potential vanilloid 1 (TRPV1) channels are primarily expressed by nociceptors (Aδ and C-fibers), where they play a crucial role in nociceptive signal transduction. Some studies have revealed that TRPV1 physically interacts with the Sigma 1 Receptor (Sig-1R), a crucial molecular target that regulates the number of TRPV1 channels localized to cell membranes without affecting channel transcription. This finding emphasizes the significance of the connection between TRPV1 and Sig-1R in the mechanism of pain. As an antagonist of Sig-1R, progesterone can downregulate the expression of TRPV1 in sensory neurons, leading to a decrease in nociceptive responses (Ortíz-Rentería et al., 2018). Enhancing the inhibitory effect of GABAergic transmission in the pain pathway is one of the important mechanisms to reduce pain transmission (Zeilhofer et al., 2012). As a metabolite of progesterone, AP could alleviate neuropathic pain by modulating T-type Ca^2+^ channels and GABA-A receptors. It has been shown that the GABA-A receptor antagonist bicuculline reverses the analgesic and antinociceptive effects of AP *in vivo*, confirming that this receptor is considered to be the primary target of AP (Huang et al., 2016). Additionally, Ayoola C et al. found that in wild-type mice, local plantar injection of epipregnanolone reduced nociception (Ayoola et al., 2014). However, it was not effective in CaV3.2 knockout mice, confirming that the blocking of the CaV3.2 T-type calcium channel in sensory neurons was involved in epipregnanolone-induced analgesia.

**FIGURE 2:**
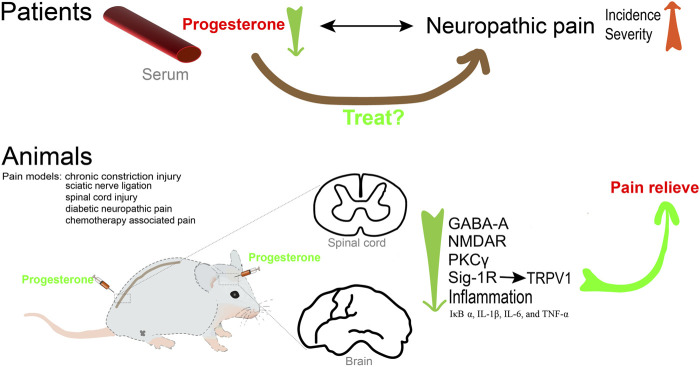
Progesterone contributes to pain relieve.

In summary (Figure 2), neuroactive steroids, including progesterone and its metabolites, play a role in pain mechanisms and deserve further attention for the development of effective steroid treatment strategies for neuropathic pain.

### Estrogen and social interaction

People who are socially isolated lack social interactions. Social isolation refers to an individual’s lack of interaction with society and the objective state of being isolated from others. Long-term social isolation is a potential source of psychological stress and is considered a significant risk factor for human illness and mortality (Brandt et al., 2022). Social interaction can have a positive impact on the physical and mental health of humans and animals by regulating the hypothalamic-pituitary-adrenal axis (HPA) axis (Devries et al., 2003). Animal studies have found that increasing social interaction can promote the recovery of neurological function in mice with cerebral ischemia (Venna et al., 2014). This suggests that promoting social interaction can be an adjunctive treatment for related diseases. Epidemiological studies have reported gender differences regarding lifetime prevalence rates, showing that social anxiety disorder affects women more frequently than men (Weinstock, 1999). In rodents, males typically display higher levels of social interest compared to females, highlighting sex differences that may naturally exist in social investigation and behavior (Rigney et al., 2021).

Studies suggest a potential role of estrogen in social behavior. E2-treated female animals displayed increased affiliative behaviors and decreased aggressive behaviors towards unfamiliar females in the social interaction test (Simon et al., 2004). Social interaction testing involves placing familiar or unfamiliar male or female rodents in a new environment and monitoring their exploration and social behavior. In addition, female voles treated with E2 exhibited a significant preference for females over males in the sexual preference test (Lian et al., 2020). When conjugated equine estrogen was administered subcutaneously to ovariectomized rats, it improved object recognition, increased the time spent on the open arms of the plus maze, and increased the time spent interacting with a conspecific (Walf et al., 2008). Phytoestrogens are estrogen analogues derived from plants and are found in high concentrations in soy. Phytoestrogens have been shown to modulate the DNA binding affinity of estrogen receptors (Kostelac et al., 2003); the study reported the significance of dietary phytoestrogens in maintaining the activity of a brain circuit that controls aggressive and social behavior in male mice. After a 6-week period of adhering to a low-phytoestrogen chronic diet, notable changes were observed in intermale aggression and territorial marking behavior. Furthermore, mice that were fed a low-phytoestrogen diet exhibited a decline in sociability and a diminished preference for social odors (Sandhu et al., 2020). These findings suggest a disruption in social behavior, which was accompanied by a significant decrease in c-Fos induction in various brain regions, including the medial and cortical amygdala, lateral septum, medial preoptic area, and the bed nucleus of the stria terminalis. Long-term effects of estrogen replacement on social investigation and social recognition in ovariectomized female mice have been documented through the use of 60-day time-release pellets containing physiological doses of E2 (Tang et al., 2005). After a treatment period of 55 days, mice that were administered the 0.72-mg pellet exhibited evidence of social recognition memory, as determined by a 24-hour habituation test.

### Mechanisms

OT serves as the neurohormonal foundation for social interaction. Studies have demonstrated that OT has a beneficial impact on social interaction through the reduction of anxiety and stress responses (Chen et al., 2016). Evidence suggests that central oxytocin mediates social cognition, social bonding, and social anxiety. In the context of human or animal interaction, the release of endogenous OT can be enhanced. Endogenous OT and sex hormone levels were examined in a sample of 199 socially anxious individuals, consisting of 51 women in the high socially anxious group and 50 women in the low socially anxious group (Schneider et al., 2021). Regression analyses revealed a significant association between elevated levels of OT and E2 in women and a decrease in both the total Liebowitz Social Anxiety Score (LSAS) and the LSAS Fear subscale score. The correlation between hormonal interaction and social anxiety scores was found to be statistically significant among women with high levels of social anxiety in the subsample. In the male population, no significant correlations were found between endogenous hormones and LSAS scores. The aforementioned findings suggest that women exhibiting elevated levels of basal OT and E2 tend to experience lower levels of anxiety in comparison to those with lower levels of OT and E2. Social interaction exhibited a positive correlation with the expression of OT receptor and vasopressin receptor mRNA in the medial amygdala and paraventricular nucleus of the hypothalamus (Murakami et al., 2011). Furthermore, the mRNAs of OT receptor and vasopressin receptor exhibited a strong positive correlation with ERα mRNA in the medial amygdala. Similarly, the mRNAs of OT and arginine vasopressin showed a significant association with ERβ mRNA in the paraventricular nucleus of the hypothalamus. These findings suggest that the OT and arginine vasopressin systems are under tight regulation by ERs. Furthermore, the levels of OT and the binding of OT receptors exhibited a significant increase following estrogen treatment in various brain regions of female rats and mice, whether they were naturally cycling or had undergone ovariectomy (Tokui et al., 2021). E2 exerts direct modulation on the OT system, encompassing OT production, receptor binding, and the impact of OT on social behavior. Additionally, it was observed that in mice, the administration of E2 resulted in an augmentation of the anxiolytic properties of OT (Mccarthy et al., 1996). Estrogen was found to mitigate the stress levels associated with social interactions. Specifically, it reduced the anxiety of mice when approaching unfamiliar mice by decreasing the concentration of corticosterone (Eid et al., 2020). Consequently, this led to an increase in the baseline frequency of social interactions.

**FIGURE 3:**
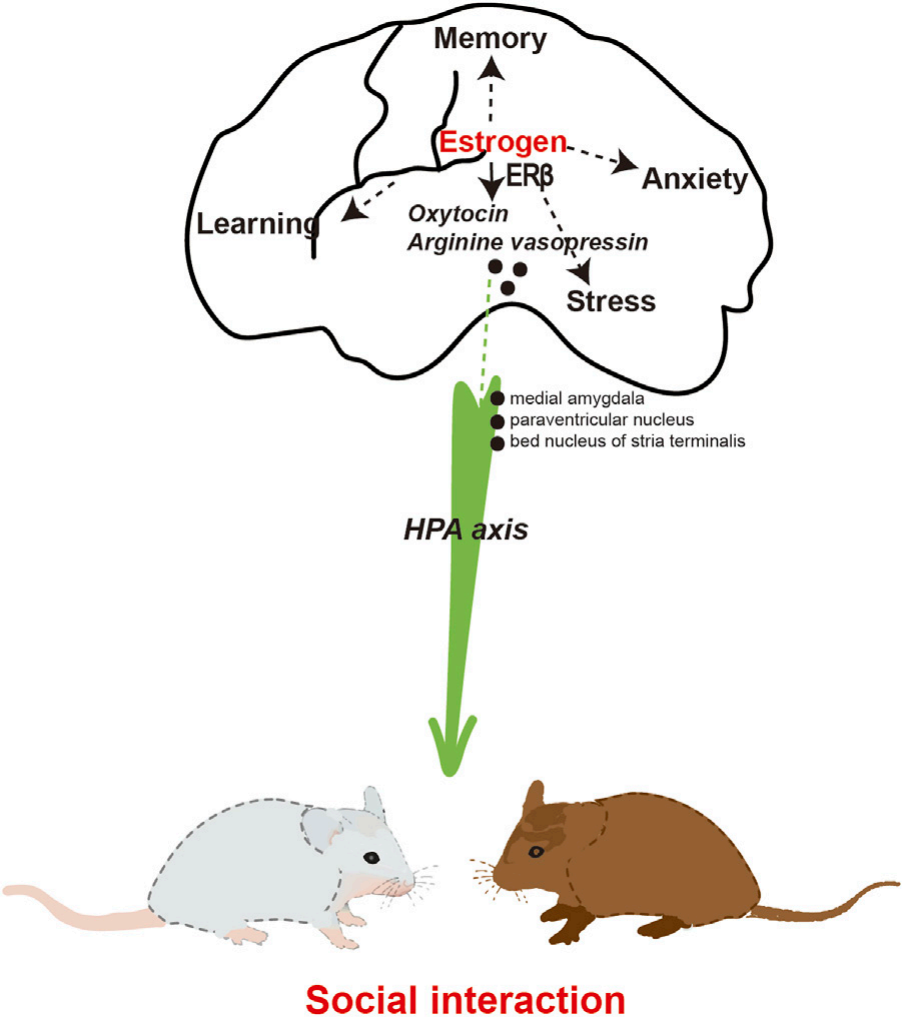
The involvement of estrogen in social interaction.

E2 facilitation of psychosocial anxiety in females may depend on the presence of ERα and ERβ. Female ERα and ERβ knockout mice exhibit impairments in social interaction, specifically in the manifestation of social anxiety (Clipperton et al., 2008; Tsuda et al., 2018). Mice that lacked the ERβ gene specifically in the CNS displayed a decrease in social interaction. This was accompanied by altered expressions of OT and arginine vasopressin in the bed nucleus of the stria terminalis (Dombret et al., 2020). ERβ knockout mice exhibited impairments in neuronal migration and eliminated the E2-induced reductions in depressive behavior observed in mice (Xu et al., 2016; Varshney et al., 2017). Additionally, the administration of an ERβ agonist has demonstrated the ability to decrease anxiety and depressive-like behavior in rats (Choleris et al., 2008). Estrogen plays a crucial role in social interaction through the activation of ERβ and the regulation of OT (Figure 3). However, the implementation of ERT for the purpose of treating or enhancing social interaction is not yet widely practiced in clinical settings.

### Progesterone and cachexia

Cachexia is a debilitating wasting syndrome that is associated with numerous chronic diseases, including cancer, acquired immune deficiency syndrome (AIDS), chronic obstructive pulmonary disease (COPD), anorexia nervosa, neurodegenerative diseases, and other terminal illnesses. It is estimated that 60%–80% of patients with advanced cancer, 25% of patients with COPD, and 28%–54% of patients with end-stage renal failure suffer from this syndrome (Wagner, 2008; Von Haehling et al., 2010; Koppe et al., 2019). Cachexia is characterized by a loss of appetite, weight loss, tissue wasting, accompanied by a decrease in muscle mass and adipose tissue. It also leads to worse tolerance to anticancer therapy, reduced quality of life (QoL), and shortened survival. For patients with cachexia, improving appetite and promoting weight gain are important treatment goals.

Two available drugs for progesterone therapy, MPA and megestrol acetate (MA) were used to treat cachexia. These drugs have shown to increase appetite and promote weight gain. The US FDA licensed MA for the treatment of anorexia, cachexia, or unexplained weight loss in patients with AIDS-associated cachexia in 1993. There is now ample evidence of progestins having a supportive effect for cachexia. The French Cancer Centre concluded that MA, as well as MPA, are appetite stimulants that are effective in controlling cancer-related cachexia and weight loss (Desport et al., 2000). The 2010 European Palliative Care Research Collaborative Cachexia Guidelines recommended that progesterone therapies “should be considered for patients with refractory cachexia and anorexia as major distressing symptoms” in cancer patients. In patients with locally recurrent or metastatic nasopharyngeal carcinoma who received MA or MPA for cachexia management, 80.5% experienced weight gain, improved QoL and pain control, and reduced plasma Epstein-Barr virus DNA load (Hung et al., 2017). A 2013 Cochrane review, which encompassed 23 RCTs and involved 3,428 cancer patients, affirmed that individuals undergoing MA treatment were more prone to experiencing enhancements in appetite, weight, and QoL (Ruiz Garcia et al., 2013). Moreover, combining MPA with other anticachectic agents has been suggested as a means of enhancing their effectiveness in treating cachexia (Madeddu et al., 2009). The combination of MPA with an inhibitor of dsRNA-dependent protein kinase, which plays a role in connecting two cachectic signaling molecules to reduce synthesis and increase degradation of skeletal muscle protein, resulted in an increase in the wet weight of the gastrocnemius muscle and blood glucose, as well as a decrease in serum triglyceride, TNF-α, and IL-6 in cancer cachexia in mice (Chen et al., 2012). MPA combined with radiotherapy can not only improve the QoL of patients, but also have an adjuvant treatment effect for certain types of cancer.

However, both the American Society of Clinical Oncology and the European Society for Clinical Nutrition and Metabolism guidelines suggest the use of MA for cachexia treatment without specifying the recommended dosage, which is a more detailed concern for clinicians. In a meta-analysis including 23 trials (3,790 patients) investigating the clinical benefits of MA in cancer patients with anorexia/cachexia, studies were divided into high-dose treatment (>320 mg/day) and low-dose treatment (<320 mg/day) (Lim et al., 2022). However, patients who received high-dose MA tended to experience weight loss rather than weight gain. The author explained that patients who received higher doses of MA may have experienced more severe cachexia. A Cochrane systematic review examined the use of MA for treating anorexia/cachexia syndrome in patients with cancer, AIDS, and other chronic diseases. The review concluded that, compared with placebo, MA improved appetite and resulted in a slight weight gain of 2.25 kg, but with more adverse events (RR 1.46; 8 studies, 638 patients) though with no significant difference in terms of mortality (Ruiz-García et al., 2018). Interestingly, when compared to no treatment or other appetite stimulants, patients treated with MA gained weight without experiencing any increase in adverse events. This research also concluded that there was no difference in weight gain between lower and higher doses. However, the study used the definition of low dose and high dose outlined in each trial, which had a large variability and weakened its findings. Another network meta-analysis included 80 RCTs with 10,579 patients (48 RCTs with 7,220 cancer patients, 23 RCTs with 2643 AIDS patients) to evaluate the efficacy and safety of pharmacological interventions for cachexia (Saeteaw et al., 2021). The study concluded that compared to placebo, corticosteroids, high-dose MA combination (≥400 mg/day, 4 cancer studies, 427 patients), MPA (3 cancer studies, 285 patients), high-dose MA (≥400 mg/day, 12 cancer studies, 1,426 patients), and androgen analogues (Androgen, 3 cancer studies, 922 patients) were significantly associated with weight gain of 6.45, 4.29, 3.18, 2.66, and 1.50 kg respectively after 8 weeks of treatment. For improving appetite, high-dose MA (combination), and Androgen significantly improved the standardized appetite score. Moreover, high-dose MA did not show a significant increase in overall and serious adverse events. Lower doses (less than 400 mg/day, 11 cancer studies, 715 patients) or low-dose combination (5 cancer studies, 263 patients) did not show any benefits in terms of weight and appetite gain. In summary, when it comes to the clinical dosage for cancer-related cachexia, high-dose MA for 8 weeks, whether combined with other pharmacological interventions or not, has the potential to increase weight and appetite gain in the treatment of cachexia.

Nevertheless, the benefits of cachexia in patients with AIDS or other pathologies were inclusive. In a review that included 38 RCTs assessing anorexia/cachexia related to various pathologies, treatment with MA resulted in a weight gain of 1.47 kg in patients with other underlying pathologies except for cancer and AIDS, compared to placebo (4 studies, 244 patients) (Ruiz-García et al., 2018). For patients with AIDS, 20 studies (2,122 participants) assessed the difference in weight gain and reported that high-dose MA and high-dose combination significantly improved weight gain, with an increase of 3.81 kg and 3.14 kg, respectively (Saeteaw et al., 2021). In a review assessing MA for non-cancer cachexia patients including12 RCTs and 6 non-randomized trials, involving 916 patients (11 AIDS studies, 607 patients), it was found that mean weight change in patients receiving MA was positive in all studies (Taylor et al., 2016). There was insufficient data to support appetite improvement. Therefore, it seems that patients with AIDS can also benefit from MA treatment, but there is a lack of evidence for its effectiveness in other pathologies such as COPD and end-stage renal failure.

### Mechanisms

The pathogenesis of cachexia is a multifactorial process mediated predominantly by proinflammatory cytokines under NF-κB control such as TNF-α, IL-1, IL-6, interferon-γ, and leukemia inhibitory factor (Camargo et al., 2015). High serum levels of proinflammatory cytokines have been reported in patients with advanced cancer-related cachexia. Increased production of IL-1, IL-6, and TNF-α was observed in phytohemagglutinin-stimulated cultured peripheral blood mononuclear cells isolated from advanced cancer patients (Mantovani et al., 2000). Moreover, chronic administration of these cytokines is capable of reproducing some features of cachexia *in vivo*, while treatment with specific antagonists can reverse their effects. Among these factors, TNF-α is the first factor confirmed to be related to cancer-related cachexia. Injection of TNF-α into experimental animals induces anorexia, weight loss, and the development of cachexia; while preemptive administration of TNF-α antibody mitigated cancer cachexia manifested with significantly less weight loss and leg muscle preservation (Kang et al., 2022). It can promote fat mobilization by inhibiting lipoprotein lipase, which encourages adipocytes to uptake fatty acids from plasma lipoproteins and convert them into triglycerides. Interferon-γ and IL-6 also have a similar effect to TNF-α on lipid metabolism in animal studies of cancer cachexia. TNF-α can inhibit protein synthesis and increase protein degradation. In addition, TNF-α and IL-1 can not only increase the capacity for glucose utilization, but also elevate glucose concentration.

The mechanism of progesterone-induced appetite stimulation and weight gain in cachexia is not fully understood. It is thought to act by inhibiting cytokines, as observed in both animal and human studies. MPA was able to abolish cachexia and reduce systemic levels of IL-6. It also suppressed TNF-α-induced IL-6 secretion. In addition, intramuscular injections of MPA into nude mice bearing KPL-4 (a human breast cancer cell line) transplanted tumors significantly decreased serum IL-6 levels without affecting tumor growth and maintained the body weight of recipient mice (Kurebayashi et al., 1999). Moreover, MPA significantly inhibits IL-6 and IL-8 promoter-reporter constructs at the transcriptional level by interfering with NF-κB and activator protein-1 in mouse fibroblast cells (Koubovec et al., 2004). These findings indicate that the suppression of proinflammatory cytokine secretion may, at least in part, contribute to the anticachectic effect of MPA.

In addition, MA can stimulate the hypothalamus to produce neuropeptides that increase appetite. Increased production of neuropeptide Y, a potent central appetite stimulant, is thought to be associated with an increase in food intake. Neuropeptides can regulate the ventromedial hypothalamic nucleus (the satiation center) by reducing the firing impulses of neurons and inhibiting the activity of pro-inflammatory cytokines, such as IL-1, IL-6, and TNF-α. MPA has been confirmed to inhibit the production of TNF-α, promote protein synthesis, and increase adipose tissue. MA can increase insulin-like growth factor (IGF-1) levels in breast cancer patients, which is presumably one of the mechanisms of weight gain caused by MA (Helle et al., 1999). This is because IGF-1 has been shown to increase appetite. By utilizing dual-energy X-ray absorptiometry to conduct a comprehensive body scan, Loprinzi discovered that MA resulted in weight gain, primarily due to a significant rise in adipose tissue, as opposed to fluid retention (Loprinzi et al., 1993). This is particularly important for cancer patients with cachexia, as the primary consequence of energy expenditure is the depletion of adipose tissue.

**FIGURE 4:**
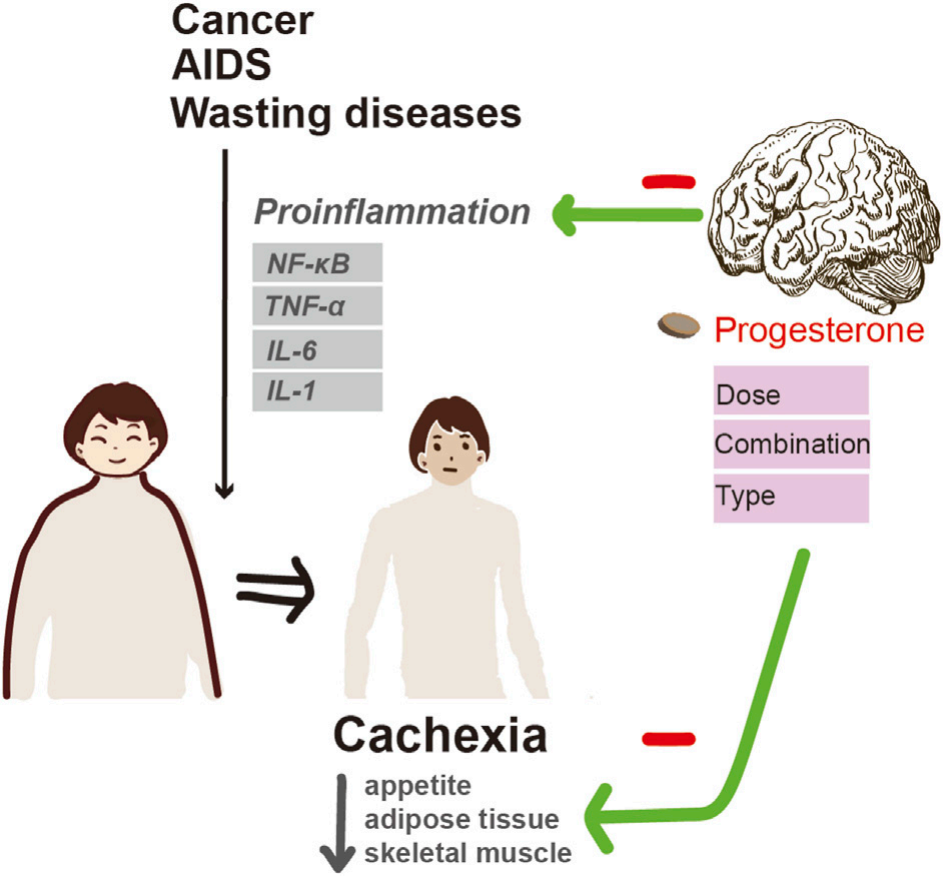
Progesterone alleviates cachexia via inhibiting inflammation and improving appetite.

In general (Figure 4), progesterone therapies have been shown to improve appetite and weight gain in cancer and AIDS patients with cachexia. However, the optimal dosage is still unclear, although there is a tendency towards high doses. For cachexia patients with other diseases, the efficacy of MA in improving weight gain and appetite has yet to be determined. Since cancer-related cachexia is a multifactorial process, it is unlikely that MA or MPA alone can counteract the complex processes involved in cachexia. Accordingly, combining MPA with other novel anticachectic agents may be a more effective way of treating cachexia.

### Safety of HRT

Progesterone is typically well tolerated among patients with advanced-stage cancer. The most commonly reported adverse events include the occurrence of peripheral edema and an elevated risk of thromboembolic complications, particularly deep vein thrombosis in the lower extremities (Marjoribanks et al., 2012). However, the latter is rarely severe. Other potential adverse events could be attributed to the glucocorticoid-like activity. It is improbable that patients with cachexia who are taking MPA will need to discontinue the medication due to the aforementioned adverse events. When HRT is administered to enhance the QoL in patients with malignancies, a crucial factor to consider is the potential for HRT especially ERT to induce tumor recurrence or metastasis (Table 2).

**TABLE 2:** Risks and benefits of estrogen treatment.

Therapy	Tumor	Risks	Benefits	Conclusion	References
ERT	Breast cancer	Augments the susceptibility to breast cancer among women		ERT should not be administered to individuals with breast cancer	Baber et al. (2016)
Estrogen and progesterone	Endometrial carcinoma	Estrogen-only therapy did not demonstrate this protective effect	Protective effect against the recurrence	Combination of estrogen and progesterone reduces the recurrence of endometrial carcinoma	Barakat et al. (2006), Shim et al. (2014)
ERT	Ovarian cancer		Improve overall patient survival	It is generally recommended to avoid ERT in patients with granulosa cell tumor	Eeles et al. (2015), Mascarenhas et al. (2006), Pergialiotis et al. (2016), Bešević et al. (2015), Del Carmen and Rice (2017)
ERT	Colorectal cancer		Decrease the risk of colorectal cancer in postmenopausal women; have a protective effect in the development and progression of intestinal tumors; reduce morbidity and mortality associated with colorectal cancer; ERβ can serve as a favorable prognostic indicator in the management of colorectal cancer	ERT exerts a beneficial impact on colorectal cancer	Murphy et al. (2015), Koch et al. (2022), Prentice et al. (2009), Peng et al. (2017)
ERT	Hepatocellular carcinoma		Reduce the incidence of hepatocellular carcinoma; inhibiting fibrosis and development of hepatocellular carcinoma	Reduce the occurrence of hepatocellular cancer and improved overall survival rates	Adami et al. (1989), Sukocheva (2018), Shi et al. (2014), Montella et al. (2015)
ERT	Gastric cancer	ER + status is a predictor of unfavorable prognosis in the treatment of gastric cancer; ERα and ERβ are prognostic indicators for gastric cancer		Avoid initiating ERT in individuals who have previously been diagnosed with gastric cancer	Zhao et al. (2003), Matsui et al. (1992), Ur Rahman and Cao (2016)
ERT	Pancreatic cancer	No significant association between the risk of pancreatic cancer and the use of ERT.		Not a contraindication for ERT.	Tang et al. (2015)
ERT	Prostate cancer	Contribute to the development of benign prostatic hyperplasia and prostate cancer		Several estrogen antagonists are currently undergoing clinical trials. Toremifene exhibited a significant improvement in the recurrence of bone metastatic prostate cancer	Fujimura et al. (2015)
ERT	Lung cancer	Tumor-promoting effect in lung cancer. ERα and ERβ are prognostic indicaters		ERT should not be administered to patients with lung cancer	Hsu et al. (2017)

## Conclusion

The regulation of female hormones on brain health, particularly their potential treatment on appetite, pain, depression, and social isolation is gaining attention. Progesterone has been found to be advantageous in promoting weight gain and stimulating appetite when used in the management of cachexia associated with cancer or AIDS. Exogenous progesterone also contributes to alleviating allodynia. Furthermore, progesterone has been authorized for the treatment of PPD. Therefore, the administration of progesterone may be considered as a treatment option for patients experiencing pain, depression, and cachexia. Estrogens are known to have an antidepressant effect and to contribute to social interaction. However, concerns have been raised regarding their potential to induce tumor recurrence or metastasis, which has hindered their clinical acceptance. Nonetheless, estrogen therapy can actually reduce the risk of colorectal cancer and hepatocellular carcinoma. Further considerations regarding the risks and benefits of estrogen therapy in breast cancer, endometrial carcinoma, ovarian cancer, gastric cancer, pancreatic cancer, prostate cancer, and lung cancer are necessary. Nevertheless, medical professionals are still unable to provide compelling answers regarding the specific selection of drugs, routes of administration, dosage, timing of initiation and duration, and the decision to use combination therapies, except for the rapid control of depression symptoms with brexanolone and zuranolone. It is worth noting that drugs with a rapid onset for severe symptoms are generally more likely to be approved by the FDA. However, the prevention or efficacy only after long-term administration of hormones is often affected by various factors and needs to be studied repeatedly. However, the potential benefits of female hormones in brain health should not be overlooked.

## Figures and Tables

**Table:** 

**AIDS**	Acquired immune deficiency syndrome
**AP**	Allopregnanolone
**CNS**	Central nervous system
**CSF**	Cerebrospinal fluid
**CRC**	Colorectal cancer
**CCI**	Chronic constriction
**COPD**	Chronic obstructive pulmonary disease
**DA**	Dopamine
**DHP**	Dihydroprogesterone
**FDA**	Food and Drug Administration
**E2**	Estradiol
**ER**	Estrogen receptor
**ERT**	Estrogen replacement therapy
**fMRI**	Functional magnetic resonance imaging
**GPER**	G protein-coupled estrogen receptor
**Dichtel et al.**	Hormone therapy
**HRT**	Hormone replacement therapy
**HPA**	Hypothalamic-pituitary-adrenal axis
**IGF-1**	Insulin-like growth factor
**LSAS**	Liebowitz Social Anxiety Score
**MPA**	Medroxyprogesterone acetate
**MA**	Megestrol acetate
**MT**	Menopausal transition
**MHT**	Menopausal hormone therapy
**NLRP3**	Nucleotide-binding and oligomerization domain-like receptor family 3
**OCPs**	Oral contraceptives
**OT**	Oxytocin
**PPD**	Postpartum depression
**PR**	Progesterone receptors
**QoL**	Quality of life
**RCTs**	Randomized controlled trials
**SSRI**	Selective serotonin reuptake inhibitors
**Sig-1R**	Sigma 1 Receptor
**TRPV1**	Transient receptor potential vanilloid 1
**5-HT**	5-hydroxytryptamine
**GABA**	γ-aminobutyric acid
